# Molecular subtyping improves breast cancer diagnosis in the Copenhagen Breast Cancer Genomics Study

**DOI:** 10.1172/jci.insight.178114

**Published:** 2024-04-08

**Authors:** Tobias Berg, Maj-Britt Jensen, Alan Celik, Maj-Lis Talman, Maria Anna Misiakou, Ann Søegaard Knoop, Finn Cilius Nielsen, Bent Ejlertsen, Maria Rossing

**Affiliations:** 1 Danish Breast Cancer Group,; 2 Department of Clinical Oncology,; 3 Center for Genomic Medicine, and; 4 Department of Pathology, Rigshospitalet, Copenhagen University Hospital, Copenhagen, Denmark.; 5 Department of Clinical Medicine, University of Copenhagen, Copenhagen, Denmark.

**Keywords:** Oncology, Breast cancer, Clinical practice, Molecular pathology

## Abstract

**BACKGROUND:**

Intrinsic molecular subtypes define distinct biological breast cancers and can be used to further improve diagnosis and risk allocation.

**METHODS:**

The Copenhagen Breast Cancer Genomics Study (CBCGS) prospectively included women diagnosed with breast cancer at Rigshospitalet from 2014 to 2021. Eligible patients were females with a primary invasive breast cancer (T1c, if N0M0; otherwise, any T, any N, or any M stage) and no prior malignancy. All patients underwent molecular profiling with the CIT256 and PAM50 molecular profile.

**RESULTS:**

In the study period, 2,816 patients were included in the CBCGS. Molecular subtyping showed an increase in nonluminal (molecular-apocrine, luminal C, and Basal-like) as compared with luminal (luminal A, luminal B, and Normal-like) subtypes with increasing stage from I to IV. Across all stages, we found a significant difference in survival among subtypes; 91% of patients with LumA were alive at 5 years compared with 91% for LumB, 84% for LumC, 82% for mApo, and 80% for Basal-like. We identified 442 tumors (16%) that were discordant in subtype between CIT256 and IHC. Discordant subtype proved to be a risk factor of death among patients with IHC luminal breast cancer (hazard ratio [HR], 2.08; 95% CI, 1.51–2.86) in a multivariable Cox regression analysis. Discordance occurred more often among patients with N3, stage IV, or grade III disease.

**CONCLUSION:**

Our findings indicate that molecular subtypes are a predominant classification for survival. Assessment is particularly crucial for patients with IHC luminal breast cancer with known high-risk factors, since they are at an increased risk of harboring an aggressive molecular subtype.

## Abstract 2

Genomic alterations in breast cancer tumors better predict survival than traditional pathological assessments.

## Introduction

Intrinsic molecular subtypes of breast cancer are biologic entities associated with specific prognostic and therapeutic features and provide further prognostic information than traditional clinical assessment with staging and receptor expression (1–3). Of the original 5 intrinsic subtypes, (Luminal A, Luminal B, HER2-enriched, Basal-like, and Normal-like) commercially available platforms have been made available based on 50- or 21-gene molecular classifiers (4, 5).

Within the traditional 3 IHC subgroups and with addition of other clinical and histologic factors, such as tumor size and grade, lymph node involvement, patients’ ages, and menopausal status, prognosis, and response to specific therapies is estimated. However, there remains a substantial difference in the behavior of breast cancers even within each one of such classifications. This discordance in breast cancer biology suggests that the true heterogeneity of epithelial breast cancers is much more vast than initially suspected.

A broad introduction of molecular classifiers into clinical practice is ongoing; however, issues have been raised due to the heterogeneity seen in ER^+^ tumors as well as the synonymous approach of Basal-like and triple-negative tumors (6, 7). The CIT256 intrinsic subtype has attempted to tackle these issues by integrative analysis and reclassification of intrinsic subtypes into 6 stable molecular subgroups that largely correspond to the original 5 subtypes, but without an ERBB2 subgroup and the separation of the large luminal ER^+^ group into 4 subgroups (8).

Metastatic development in breast cancer can be seen as either occurring due to diagnostic delay (either by patient or system) or due to clinicomolecular aggressive factors (9–11). Previous studies utilizing IHC subgroups as a surrogacy for intrinsic molecular subtypes have indicated a rise in aggressive nonluminal subtypes with increasing stage (12–14). Similarly, in primary metastatic tumors, IHC subgroups have been shown to be a risk factor for distant metastases in T1 tumors but not in T3/T4 tumors; this reflects the hypothesis that small tumors may harbor a potentially unavoidable systemic dissemination dependent on subtype. However, if that does not occur, continuous extension will lead to metastatic disease, regardless of subtype (15).

The Copenhagen Breast Cancer Genomics Study (CBCGS) was initiated in 2014 and provides unique insight in how molecular subtypes may be part of a standard of care diagnostic pipeline (16). In this study, we examine if there is a shift in intrinsic molecular subtypes across stages and tumor size in a consecutive breast cancer cohort. Furthermore, we wish to examine the clinically relevant information provided by intrinsic molecular subtypes, how it differs from IHC subgroups, and whether it may guide future treatment decisions.

## Results

### Patient demographics and flowchart.

**Figure 1:**
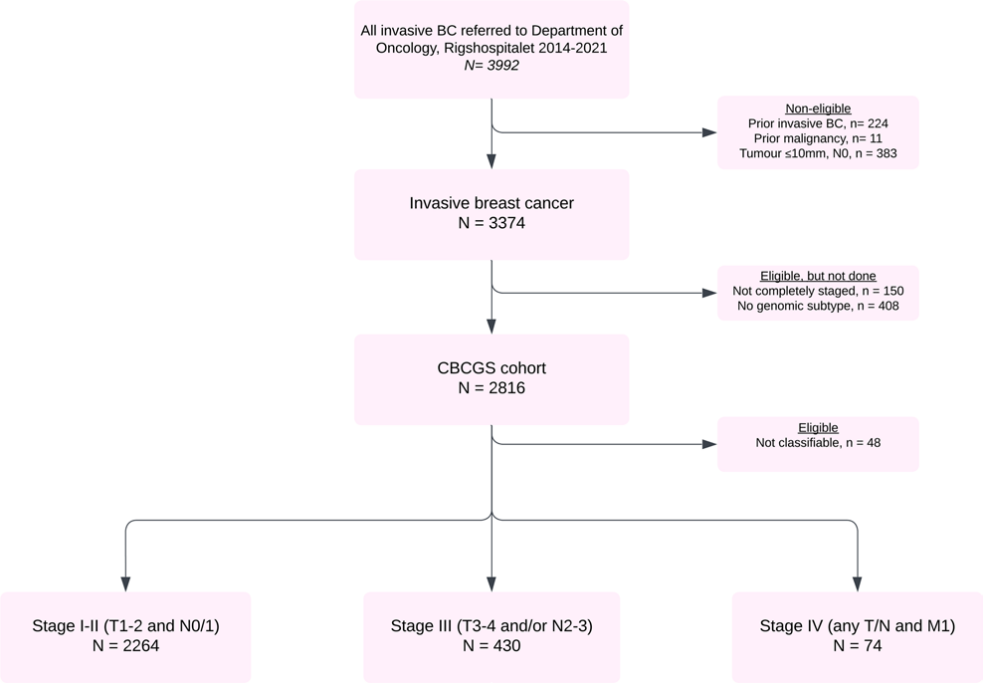
Flow diagram of patients diagnosed at Rigshospitalet from 2014 to 2021. BC, breast cancer; CBCGS, Copenhagen Breast Cancer Genomics Study.

Between April 2014 and December 2021, 3,992 women were diagnosed with an invasive breast cancer at Copenhagen University Hospital, Rigshospitalet, and were referred to the Department of Oncology. Among the 3,768 women with a first invasive breast cancer, 383 had a tumor smaller than 10 mm and 11 had prior malignancy. Of the 3,374 eligible patients, 408 (12.1%) were not subtyped and 150 (4.4%) were biopsied with no further diagnostic workup. Thus, 2,816 women (83.5%) were included in the CBCGS cohort (Figure 1): 2,300 in stage I/II (81.7%), 442 in stage III (15.6%), and 74 in stage IV (2.7%). In total, 48 patients could not be classified by CIT256 and/or PAM50 (outliers) and were excluded from further analyses.

Table 1 shows patient and tumor characteristics. A stage-by-stage increase in HER2-positivity is seen (12%–30%) and an increase in ER^–^ disease from stage I–III versus IV (12%, 17%, and 15% versus 26%, respectively), was identified. The CIT256 subtype differed significantly across stages. mApo increased from 5.1% in stage I to 14% in stage IV and Lumincal C (LumC) from 11% in stage I to 26% in stage IV; however, the same trend was not evident for Basal-like. A similar pattern was seen for PAM50.

### Addition of molecular subtype at diagnosis.

To investigate trends in molecular subtypes, stage, and tumor size, Table 2 demonstrates the distribution of CIT256 subtype, PAM50 subtype, and IHC subtype by stage (III and IV) and tumor size. No apparent difference was evident as to the classification of luminal versus nonluminal, whether it is examined by molecular subtyping scheme (CIT256; PAM50) or IHC. We did see a higher representation of nonluminal tumors in stage IV as compared with stage III and a shift toward more nonluminal tumors with increasing tumor stage — especially seen in stage IV with 69% nonluminal tumors in T3/T4. LumA was highly represented in T1 tumors in both CIT256 and PAM50, with an intermediate representation in T2 falling to almost half in T3/T4. We saw a higher representation of LumC, mApo, and HER2-enriched tumors in T3/T4 parallel to the increase of HER2^+^ tumors by stage (Supplemental Tables 1 and 2; supplemental material available online with this article; https://doi.org/10.1172/jci.insight.178114DS1). The same was not evident for Basal-like tumors.

**Figure 2:**
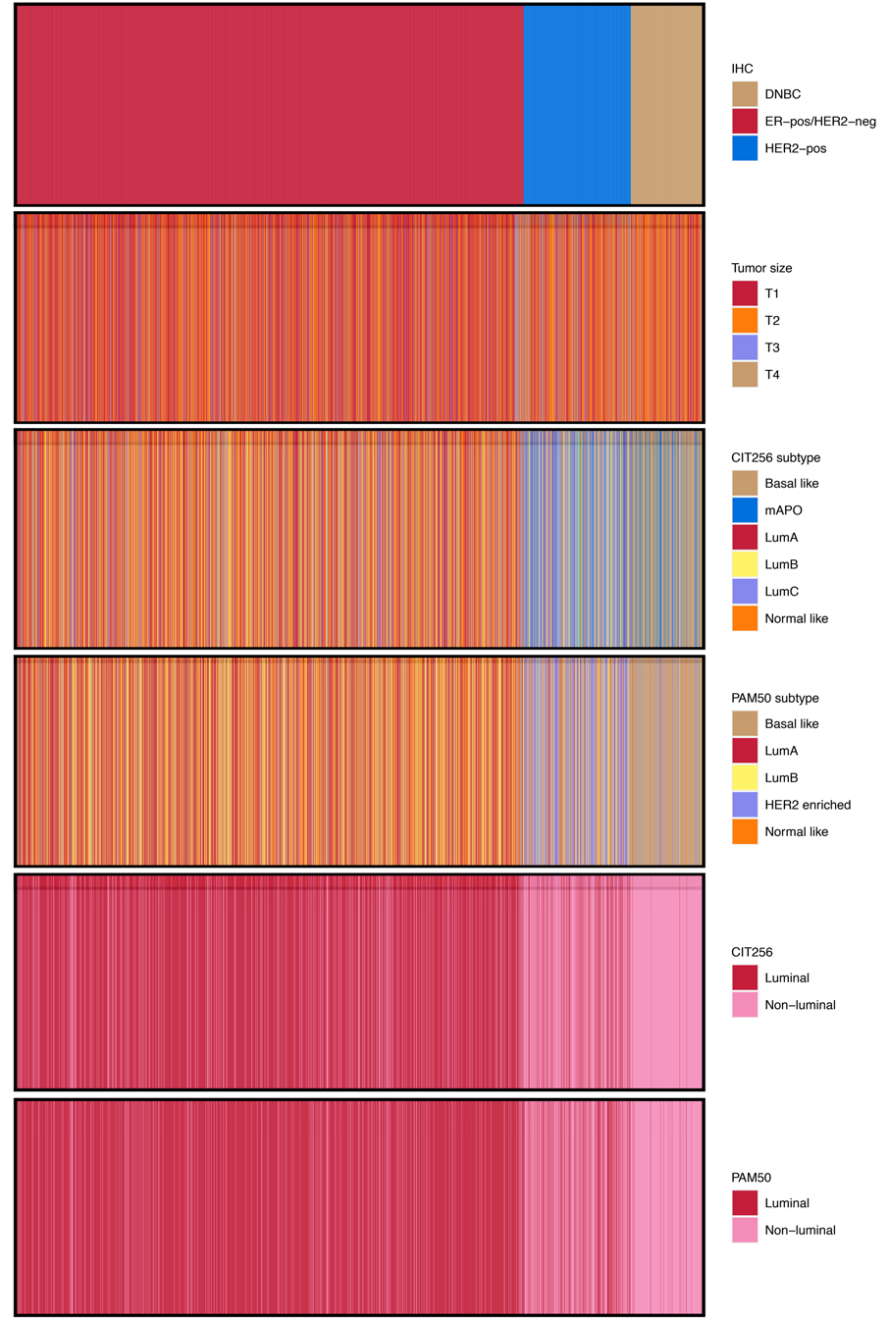
Visual correlation of tumor characteristics. Stacked Visual correlation of tumor characteristics of IHC, tumor size, and molecular subtypes (*n* = 2,768). Each line from top to bottom represents a patient.

The associations between IHC, tumor size, and intrinsic subtypes are depicted in (Figure 2). No obvious associations are seen between IHC subtype and tumor size, nor in intrinsic subtypes. Overall trends indicate that most patients are classified correctly by crude IHC classification in luminal versus nonluminal subtypes. However, we found that 442 (16%) tumors were discordant, comparing IHC Lum versus nonluminal and CIT256 Lum versus nonluminal; discordance was especially evident among the 429 IHC HER2^+^ tumors, where 147 (34%) were assigned a luminal CIT256 subtype (185 for PAM50). Supplemental Figure 1 shows the correlation between PAM50 and CIT256 subtypes. In total, 91% of luminal PAM50 tumors are also luminal on CIT256; 94% of nonluminal PAM50 tumors are also nonluminal on CIT256.

### Survival.

**Figure 3:**
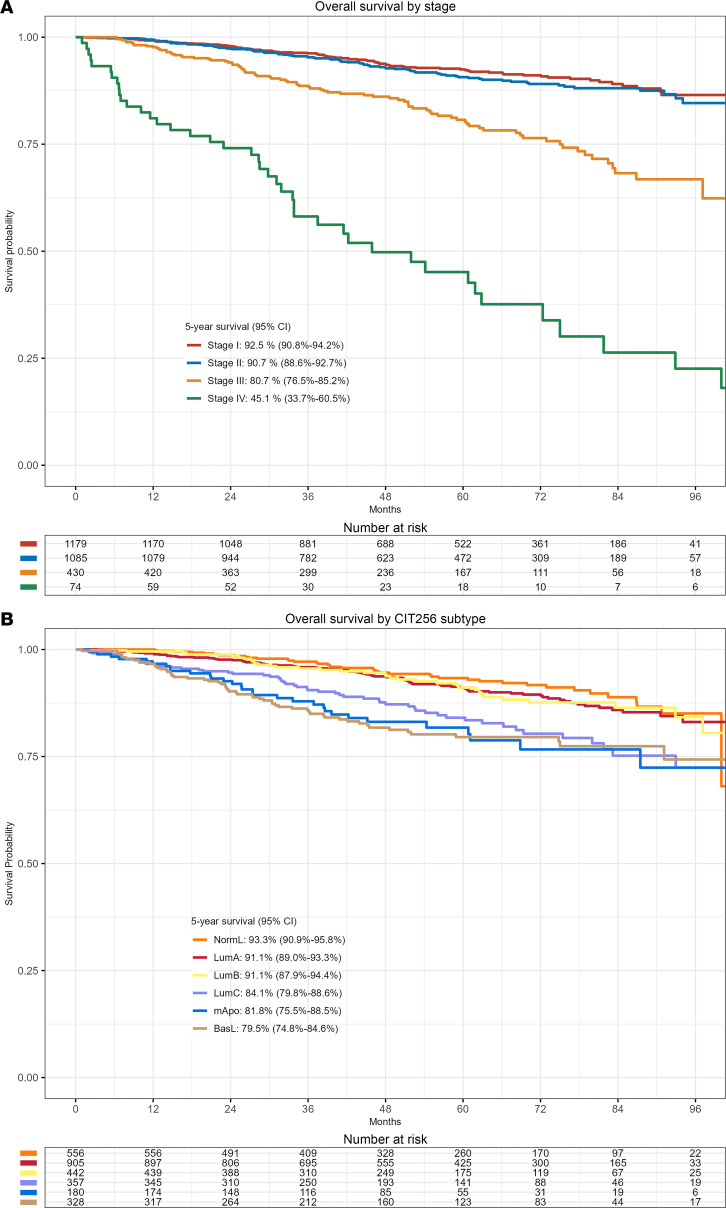
Survival curves. (**A**) Kaplan-Meier survival curve of overall survival by stage at diagnosis (*n* = 2,768). (**B**) Kaplan-Meier survival curve of overall survival by CIT256 subtype (*n* = 2,768).

Estimated median potential follow-up was 57.9 months, with 308 deaths registered. Overall survival by stage shows a deteriorating survival probability with increased stage (*P* < 0.0001). Five-year survival was 92.5% (95% CI, 90.8%–94.2%), 90.7% (95% CI, 88.6%–92.7%), 80.7% (95% CI, 76.5%–85.2%), and 45.1% (95% CI, 33.7%–60.5%) for stage I, II, III, and IV, respectively (Figure 3A). Overall survival was significantly different among CIT256 subtypes (*P* < 0.0001); 91.1% (95% CI, 89.0%–93.3%) of patients with LumA were alive at 5 years compared with 91.1% (95% CI, 87.9%–94.4%) for LumB, 84.1% (95% CI, 79.8%–88.6%) for LumC, 81.8% (95% CI, 75.5%–88.5%) for mApo, and 79.5% (95% CI, 74.8%–84.6%) for Basal-like (Figure 3B). Supplemental Figure 2 shows 5-year survival estimates by stage and intrinsic molecular subtype.

### Importance of IHC and CIT256 subtype.

Among the 2,041 patients presenting with an IHC luminal tumor, 1,751 (85.8%) were, by CIT256, assigned a luminal subtype and 290 (14.2%) were assigned a nonluminal CIT256 subtype. Of the 727 patients with a nonluminal tumor by IHC, 575 (79.1%) were, by CIT256, assigned a nonluminal subtype, while 152 (20.9%) patients were assigned a luminal CIT256 subtype. The association between overall survival and subtype assignment by IHC and CIT256 was investigated in a multivariable Cox regression analysis including stage, age, IHC subtype, and CIT256 subtype (Table 3). Malignancy grade and histological subtype did not reach significance in univariable models and were not included. Compared with patients with concurring luminal IHC and CIT256 subtype (reference), a significantly higher mortality was detected in patients with dual nonluminal (IHC and CIT256) subtype (hazard ratio [HR], 2.50; 95% CI, 1.93–3.25) and in patients with a nonluminal CIT256 but with a luminal IHC subtype (HR, 2.08; 95% CI, 1.51–2.86). In contrast, overall survival was not significantly decreased in patients with luminal CIT256 but with a nonluminal IHC subtype (HR, 0.78; 95% CI, 0.40–1.54). This establishes CIT256 subtypes as being predominant for overall survival compared with IHC. This is further confirmed comparing a model with age, stage, and IHC subtype (*P* ≤ 0.001) with the model including age, stage, CIT256 subtype (*P* ≤ 0.001), and IHC subtype (*P* = 0.55). No interaction was detected between stage and IHC subtype.

Table 4 displays differences in baseline characteristics for discordant and concordant IHC luminal tumors. Discordance was most pronounced in grade III, N3, and stage IV disease with 53.2%, 44.0%, and 27.0% of cases, respectively. To investigate this further, a logistic regression model was developed (Supplemental Table 3). This model incorporated age, stage of disease, grade, and lymph node metastases and shows an increased risk of discordance for patients with grade II, III, stage IV, N3 disease, or patients younger than 60. By applying our logistic model, we identified 18 individual risk groups in our cohort, and with a cut point of 14.2%, corresponding to the proportion of tumors with discordance among IHC luminal tumors in total, the model correctly classifies 68% of all samples corresponding to a sensitivity of 59% and a specificity of 70% (Supplemental Table 4) .

## Discussion

The prospective CBCGS confirms the prognostic importance of stage at diagnosis of breast cancer with 5-year survival rates decreasing from 92.5% in stage I to 45.1% in stage IV. Across stages, we found a significant variation in the assignment of both IHC-based and intrinsic subtype with a decrease of luminal subtype with increasing stage and a particular clear distinction from stage III to IV.

We identified a large group of patients with luminal — i.e., ER^+^HER2^–^ — breast cancer, who by CIT256 were assigned a nonluminal intrinsic subtype; these patients had a significantly impaired overall survival (HR, 2.08; 95% CI, 1.51–2.86) compared with patients with a dual (IHC and CIT256) luminal subtype. However, as compared with a dual luminal subtype, we found no significant difference in overall survival among patients assigned a luminal subtype by CIT256 and a nonluminal subtype by IHC (HR, 0.78; 95% CI, 0.40–1.54). Likewise, CIT256 remained statistically significant for overall survival in the model that included both CIT256 and IHC subtypes.

To better clarify which patients might be luminal by IHC but assigned a nonluminal intrinsic subtype, we developed a logistic model to help identify patients with potential discordant tumors. We found that age younger than 60 years (OR, 1.60; 95% CI, 1.22–2.10), stage IV (OR, 2.91; 95% CI, 1.30–6.14), grade II (OR, 2.56; 95% CI, 1.81–3.70), grade III (OR, 15.5; 95% CI, 9.36–26.0), and N3-disease (OR, 1.96; 95% CI, 1.07–3.46) were all risk factors for discordance. This accuracy of the risk model is 69% using a cut-point of 14.2% risk of discordance.

The current performance of stratification for early breast cancer treatment is based on stage; histopathological factors, including receptor status; and, for some patients, biomarker assays (4, 5, 17, 18). Our results could point toward incorporating intrinsic molecular subtypes as an element in risk stratification for all patients with breast cancer, as our results indicate that CIT256 better explains survival across all stages than does IHC, as IHC alone does not capture the genomic profile in tumors.

Progression of disease from stage I to IV can essentially be attributed to aggressive clinicomolecular risk factors or diagnostic delay. Our results indicate that diagnostic delays most likely are the main contributing factor for breast cancers being diagnosed in stage I–III, as a slight, nonlinear change in nonluminal subtypes is observed from stage I to III (26%, 35%, 33%, respectively) and then clinicomolecular factors in stage IV with 53% nonluminal tumors.

Our study has several strengths. The prospective inclusion of patients and unrestricted inclusion criteria limit the risk of selection bias in our cohort. The total number of included patients also gives certain strength to the study, especially regarding patients with stage I and II disease. The inclusion of patients across all stages also allowed us to examine differences in subtypes at time of diagnosis.

A limitation in our study is the number of patients diagnosed at Rigshospitalet who appear to have fulfilled our inclusion criteria but on whom we do not have a CIT256 intrinsic subtype. This is particularly evident for stage IV disease, which only accounts for 2.6% of our cohort. We are also limited by the patients undergoing neoadjuvant therapy, on whom we do not have a complete diagnostic workup regarding their lymph node statuses. We are also limited since these patients were exclusively treated according to their IHC subtype and not molecular subtype; this may influence any conclusions on outcomes. Furthermore, our models could be further enhanced by incorporation of the individual intrinsic subtype if more patients with nonluminal subtypes were available. Inclusion of Ki-67 was not done, as it was not readily available for all patients, and we have previously shown that Ki-67 is not optimal for identification of low- and high-risk patients compared with mRNA (16).

To our knowledge, cross-stage comparison of molecular subtypes has not been published on a scale as large as ours. The primary focus of molecular subtyping has primarily been on identifying patient subgroups who would benefit from a specific treatment or strategy. Among patients with early breast cancer, most studies have examined intrinsic subtypes in stage I–II disease (19–28), some of which have restricted their samples to ER^+^ tumors, resulting in assignment of more than 90% of tumors to a luminal intrinsic subtype (19–21, 23, 25–27). However, in cohorts unselected by ER, the proportion with an intrinsic luminal subtype has been around 80%, corresponding to our results (22, 24, 28). A few studies have included stage III tumors with proportions of stage III varying from 8% to 25% and nonluminal subtypes varying from 32% to 50% overall (29–35). Some of these studies also included PAM50 subtype by stage, with stage III differing from 19% to 57% of tumors being nonluminal (31, 32, 34, 35). A Swedish study on stage IV (mainly recurrent metastatic disease) identified 57% to be nonluminal, which is comparable to our results (36). Other cohorts have recognized an increasing trend toward nonluminal A subtypes with increasing tumor size and lymph node metastases; this differs from our results but, likewise, shows that intrinsic subtypes are a stronger prognostic indicator for outcomes than IHC subtypes (34, 37). Discordance between IHC and molecular subtyping by PAM50 has previously been reported as a risk factor for an event with discordance rates varying from 10% to 38% (21, 30, 34). As a surrogate for molecular subtypes, some de novo stage IV cohorts have reported comparable rates of ER negativity and HER2 positivity, akin to our findings (38–40). However, others have identified rates of more than a third of patients presenting as either ER^–^ or HER2^+^ (41, 42). A recent study found a small improvement in predictive modeling by combining PREDICT with intrinsic subtypes, especially for patients with ER^+^ tumors, but it questions the economic burden to justify a broad implementation (43).

Intrinsic molecular subtyping provides clinically meaningful information for diagnostic workup and treatment considerations. Our results indicate that, without intrinsic molecular subtyping, more than 15% of patients are insufficiently classified by IHC alone, and molecular subtypes are a predominant classification for survival. This should serve as compelling evidence for the inclusion of molecular subtyping in the assessment of patients with breast cancer. Further trials are needed to establish how to optimize the evaluation and treatment of patients with either discordance between IHC and molecular subtype or with a low-stage, high-risk subtype.

## Methods

### Sex as a biological variable.

Only women were included, as female breast cancer accounts for more than 99% of cases of breast cancer (44).

### Study population.

The CBCGS prospectively enrolled women aged 18 or older diagnosed with primary invasive breast cancer (T1c, if N0M0; otherwise, any T, N, and M stage) at the Department of Oncology, Copenhagen University Hospital, Rigshospitalet, between April 2014 and December 2021. The diagnostic workup has previously been described, but in short, all tumor tissue from biopsies and surgical specimens was collected and analyzed prospectively (16). Detailed information on diagnosis, genomic profiling, treatment, and follow-up was registered in the clinical Danish Breast Cancer Group (DBCG) database (www.dbcg.dk). Patients were recommended treatment according to national guidelines respecting stage at diagnosis.

### Pathology.

Standard histopathological evaluation included tumor size, histological type according to WHO, and grade as defined by Elston and Ellis (45, 46). Resection margins, invasion into skin or deep fascia, lymphovascular invasion, number of axillary lymph nodes identified, and number of metastatic nodes (macro- and micrometastatic and isolated tumor cells) was likewise evaluated. ER was assessed by IHC using a cut-off point of > 1% for ER^+^ tumors, and scoring of HER2 was performed according to national guidelines (47–49).

### Assessment of CIT256 and PAM50 subtype.

Fresh pretreatment or postsurgical breast biopsies (pretreatment in case of neoadjuvant treatment) were collected in RNAlater stabilization solution (Thermo Fisher Scientific), and total RNA was isolated as previously described (50). For the majority of samples, gene expression was measured using RNA microarrays (using Human Genome U133 Plus 2.0 Array; Affymetrix). For a subset of samples, expression was quantified using next-generation sequencing (RNA-Seq) — specifically, paired-end read sequencing (2 × 125 bp) on the Illumina HiSeq2500 platform. The library preparation, data preprocessing, and molecular subtype allocation has previously been described in detail (16, 51, 52). For the CIT256 scheme, 1 of 6 subtypes (Basal-like, mApo, LumA, LumB, LumC, Normal-like) was assigned to each sample by the CIT256 tool using a distance-to-centroid approach relying on expression of 375 probe sets (8, 51). For the PAM50 molecular subtyping scheme, for RNA-Seq samples, log_2_-transformed normalized expression values were used as input for the original predictor developed by Parker et al. (4). The classifier calculated Spearman’s rank correlation between each sample and each subtype centroid for the 50 genes of interest and assigns the class (LumA, LumB, HER2-enriched, Basal-like, Normal-like) of the most highly correlated centroid to each sample. For microarray normalized expression values, the *genefu* R package was used for assigning a PAM50 subtype based on the Pearson correlation to the PAM50 centroids.

For CIT256 and PAM50, LumA, LumB, and Normal-like are referred to as “luminal,” and LumC, mApo (CIT256), HER2-enriched (PAM50), and Basal-like are referred as “nonluminal.” For IHC, ER^+^ and HER2^–^ are luminal and any HER2^+^ or double-negative breast cancer are “nonluminal.” This was chosen to reflect the clinical application of “luminal breast cancer” and due to lack of patients especially in stage III and IV.

### Staging.

Anatomic staging was based on the eighth edition of the AJCC (53). In short, patients with pT1-2, pN0-1, cM0 were stage I–II; patients with pT3-4, pN2-3, cM0 were stage III; and patients with any-T, any-N, cM1 were stage IV (pMBC). Staging on patients allocated to neoadjuvant therapy was based on ultrasound or, if not present, MRI. Thus patients with cT1-2, cN0-1, cM0 were stage I–II and those with cT3-4, cN2-3, cM0 were stage III.

### Statistics.

Patient demographics and disease characteristics were described with numbers and percentages for categorical variables and median ± IQR for age. Any difference was examined with a 2-tailed unpaired *t* test for age and χ^2^ or Fisher’s exact test for categorical variables, excluding unknowns. Overall survival was defined as time from diagnosis until death of any cause and was estimated using the Kaplan-Meier method. Groups were compared by log-rank test. Patients were censored March 1, 2023. Potential median follow-up was calculated by Schemper and Smiths’ method of reverse Kaplan-Meier (54). A multivariable Cox proportional hazards regression model was applied to assess hazard ratio of death for stage (individual stages), age (continuous), IHC subtype, and CIT subtype combined. Grade and histological subtype were, in a univariable model, found not significant (*P* > 0.1) and were not included in the final multivariable model. The proportional hazard assumption was tested by Schoenfeld residuals. Interaction between stage and IHC subtype was examined with a likelihood ratio test. A logistic regression model was applied to assess risk of discordance among patients with IHC luminal breast cancer adjusting for age (<60 versus ≥60 years), stage (stage I–III versus IV), malignancy grade (excluding unknowns), and nodal status (N0–N2 versus N3). The logistic regression model was performed including unknowns with similar results. Groups were combined using similar odds ratios (i.e., N0, N1, and N2 combined) to derive the most clinically applicable model. All tests were 2-sided, and a *P* value of < 0.05 was considered statically significant. All statistical analysis were performed using RStudio.

### Study approval.

All participants provided written, informed consent before clinical and biomarker study data were entered in the CBCGS database hosted by DBCG. Since the study did not include any contact with patients nor did it include additional use of biological material, the need to obtain a reconsent from participants for this subanalysis was waived by the Ethical Committee of the Capital Region of Denmark. In compliance with Danish regulations, the CBCGS database was authorized by the Danish Data Protection Agency (2012-58-0004, 30-1504 I-Suite 03845), and the study was approved by the Danish Breast Cancer Group (jr.no. DBCG-2015-14). Furthermore, this register-based study was reported to the Capital Regions Research Overview (P-2020-861), approved by the Capital Regions Chart Data Unit (R-22036280).

### Data availability.

All clinical data and molecular subtypes used in this study were obtained from the CBCGS data repository hosted by DBCG (www.dbcg.dk). Raw data have previously been made publicly available: microarray data reposited in GEO (GSE231629 and GSE196723) and RNA-Seq data in Zenodo (10.5281/zenodo.7898803). Data used for generation of tables and supplemental tables are not publicly available, due to institutional restrictions. The data set can be made available to qualified researchers through application to the Danish Breast Cancer Group. Please contact dbcg.rigshospitalet@regionh.dk. Values for all figures and supplemental figures can be found in the Supporting Data Values file.

## Author contribution

TB contributed with designing the study, acquiring data, analyzing data, formal statistical analysis, and writing and approving the manuscript. MBJ contributed with analyzing the data, formal statistical analysis, and writing and approving the manuscript. AC contributed with acquiring data, analyzing data, and writing and approving the manuscript. MLT contributed with acquiring data, providing reagents, and writing and approving the manuscript. MAM contributed with acquiring data, and writing and approving the manuscript. ASK contributed with designing the study, analyzing the data, and writing and approving the manuscript. FCN contributed with acquiring the data, and writing and approving the manuscript. BE contributed with designing the study, analyzing the data, and writing and approving the manuscript. MR contributed with designing the study, acquiring data, analyzing the data, providing reagent, and writing and approving the manuscript.

## Supplementary Material

Supplemental data

ICMJE disclosure forms

Supporting data values

## Figures and Tables

**Table 1:**
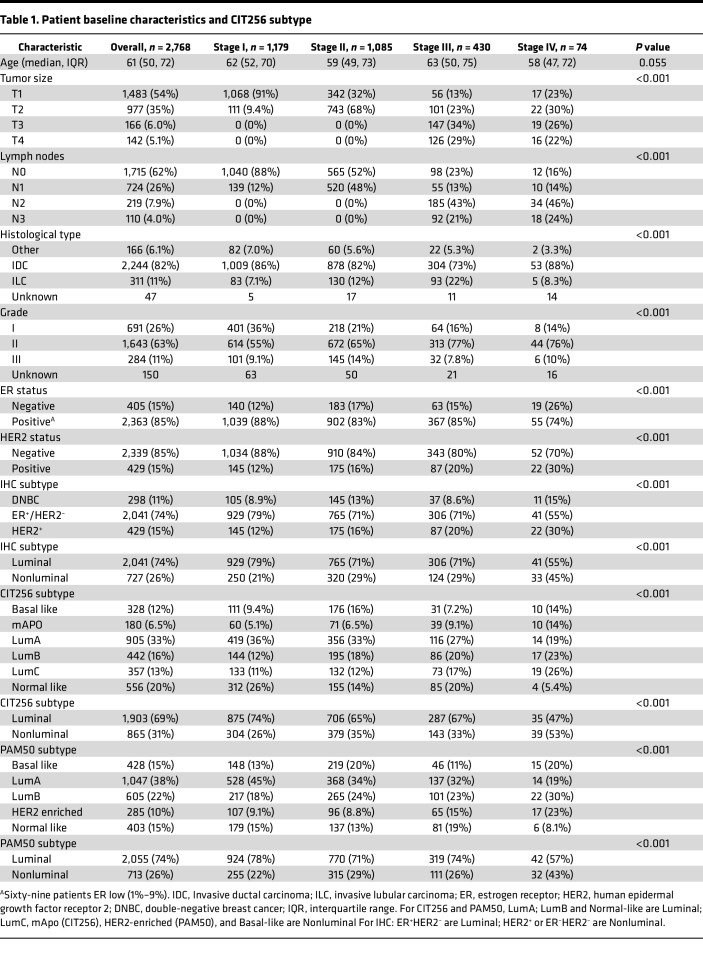
Patient baseline characteristics and CIT256 subtype

**Table 2:**
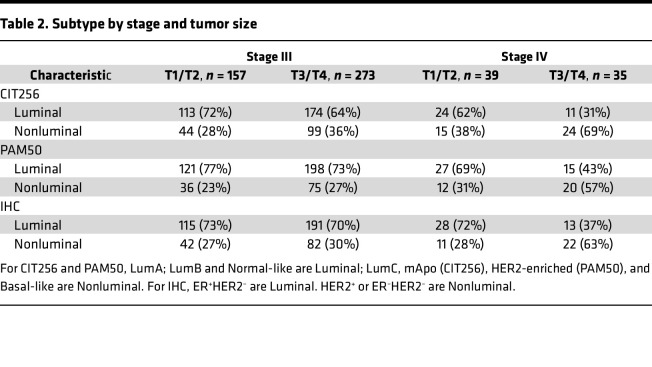
Subtype by stage and tumor size

**Table 3:**
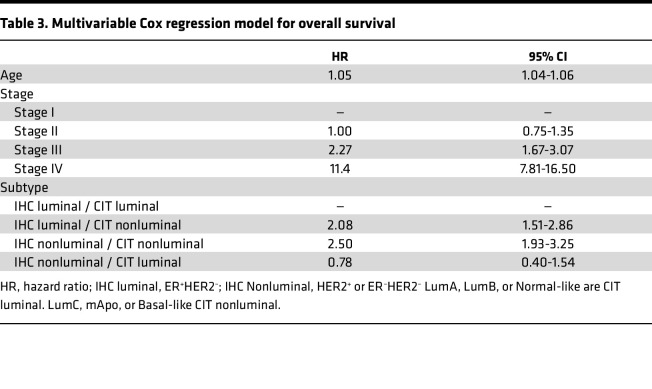
Multivariable Cox regression model for overall survival

**Table 4:**
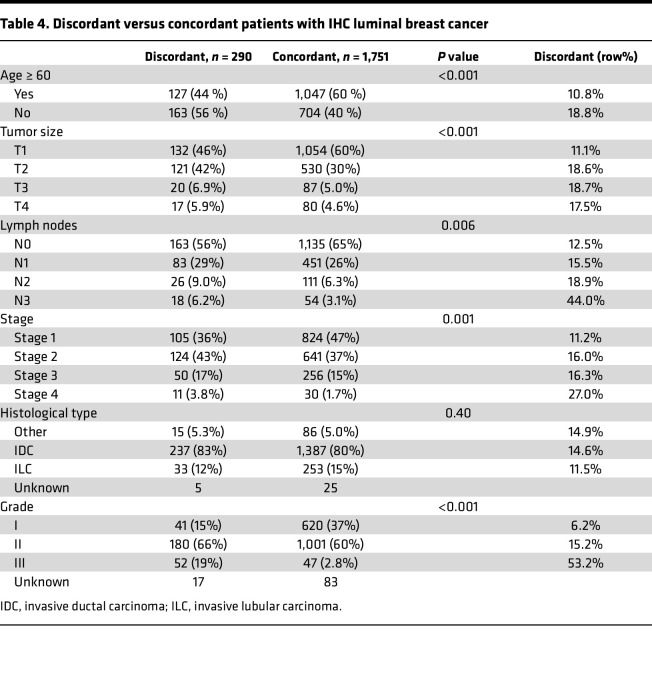
Discordant versus concordant patients with IHC luminal breast cancer

## Version 1 04/08/2024

Electronic publication
