# Supplementary material for: Molecular subtyping improves breast cancer diagnosis in the Copenhagen Breast Cancer Genomics Study
Source: JCI Insight. 2024 Apr 8;9(7):e178114. doi: 10.1172/jci.insight.178114 (PMC11128195; doi:10.1172/jci.insight.178114)

Supplementary table 1 – CIT256 subtype by tumour size

**Characteristic**	**LumA**, N = 905	**LumB**, N = 442	**Normal like**, N = 556	**Basal like**, N = 328	**mAPO**, N = 180	**LumC**, N = 357	**p-value**
**Tumour size**							<0.001
T1	566 (38%)	186 (13%)	375 (25%)	131 (8.8%)	70 (4.7%)	155 (10%)	
T2	276 (28%)	200 (20%)	115 (12%)	168 (17%)	76 (7.8%)	142 (15%)	
T3	33 (20%)	27 (16%)	36 (22%)	20 (12%)	18 (11%)	32 (19%)	
T4	30 (21%)	29 (20%)	30 (21%)	9 (6.3%)	16 (11%)	28 (20%)	

Supplementary table 2 – PAM50 subtype by tumour size

**Characteristic**	**LumA**, N = 1,047	**LumB**, N = 605	**Normal like**, N = 403	**Basal like**, N = 428	**HER2 enriched**, N = 285	**p-value**
**Tumour size**						<0.001
T1	689 (46%)	290 (20%)	217 (15%)	175 (12%)	112 (7.6%)	
T2	277 (28%)	244 (25%)	125 (13%)	212 (22%)	119 (12%)	
T3	38 (23%)	36 (22%)	38 (23%)	22 (13%)	32 (19%)	
T4	43 (30%)	35 (25%)	23 (16%)	19 (13%)	22 (15%)	

Supplementary table 3 – logistic regression model, odds of discordance in IHC luminal patients.

	**OR**	**95% CI**	**p-value**
**Age ≥ 60**			
Yes	—	—	
No	1.60	1.22-2.10	<0.001
**Stage**			
Stage I-III	—	—	
Stage IV	2.91	1.30-6.14	0.006
**Grade**			
I	—	—	
II	2.56	1.81-3.70	<0.001
III	15.5	9.36-26.0	<0.001
**Lymph nodes**			
N0-2	—	—	
N3	1.96	1.07-3.46	0.024

OR: Odds Ratio, CI: Confidence Interval.
Excluded patients with unknown malignancy grade (n=100)

Supplementary table 4 – risk groups with modelled risk. Dotted line represents suggested cutpoint. G: Grade

	Risk %	N		Observed concordant	Observed discordant
≥ 60, Stage I-III, G1, N0-2	5.0	400	**Predicted concordant**	1174	111
< 60, Stage I-III, G1, N0-2	7.7	245			
≥ 60, Stage I-III, G1, N3	9.3	5			
≥ 60, Stage I-III, G2, N0-2	11.8	628			
≥ 60, Stage IV, G1, N0-2	13.2	3			
< 60, Stage I-III, G1, N3	14.1	4			
< 60, Stage I-III, G2, N0-2	17.6	476	**Predicted discordant**	494	162
< 60, Stage IV, G1, N0-2	19.6	3			
≥ 60, Stage I-III, G2, N3	20.8	31			
≥ 60, Stage IV, G1, N3	23.0	1			
≥ 60, Stage IV, G2, N0-2	28.0	8			
< 60, Stage I-III, G2, N3	29.6	20			
< 60, Stage IV, G2, N0-2	38.4	12			
≥ 60, Stage IV, G2, N3	43.3	3			
≥ 60, Stage I-III, G3, N0-2	44.8	36			
< 60, Stage IV, G2, N3	55.0	3			
< 60, Stage I-III, G3, N0-2	56.5	61			
< 60, Stage I-III, G3, N3	71.8	2			

Supplementary figure 1 – Sankey diagram of the correlation between PAM50 and CIT256

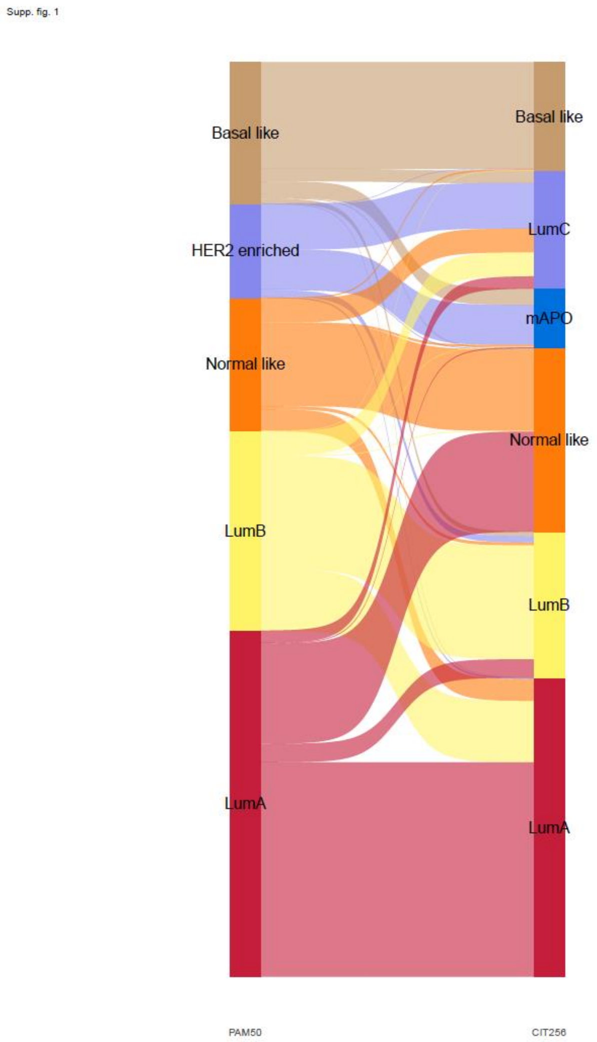

Supplementary figure 2 -5-year survival rates by stage and intrinsic molecular subtype

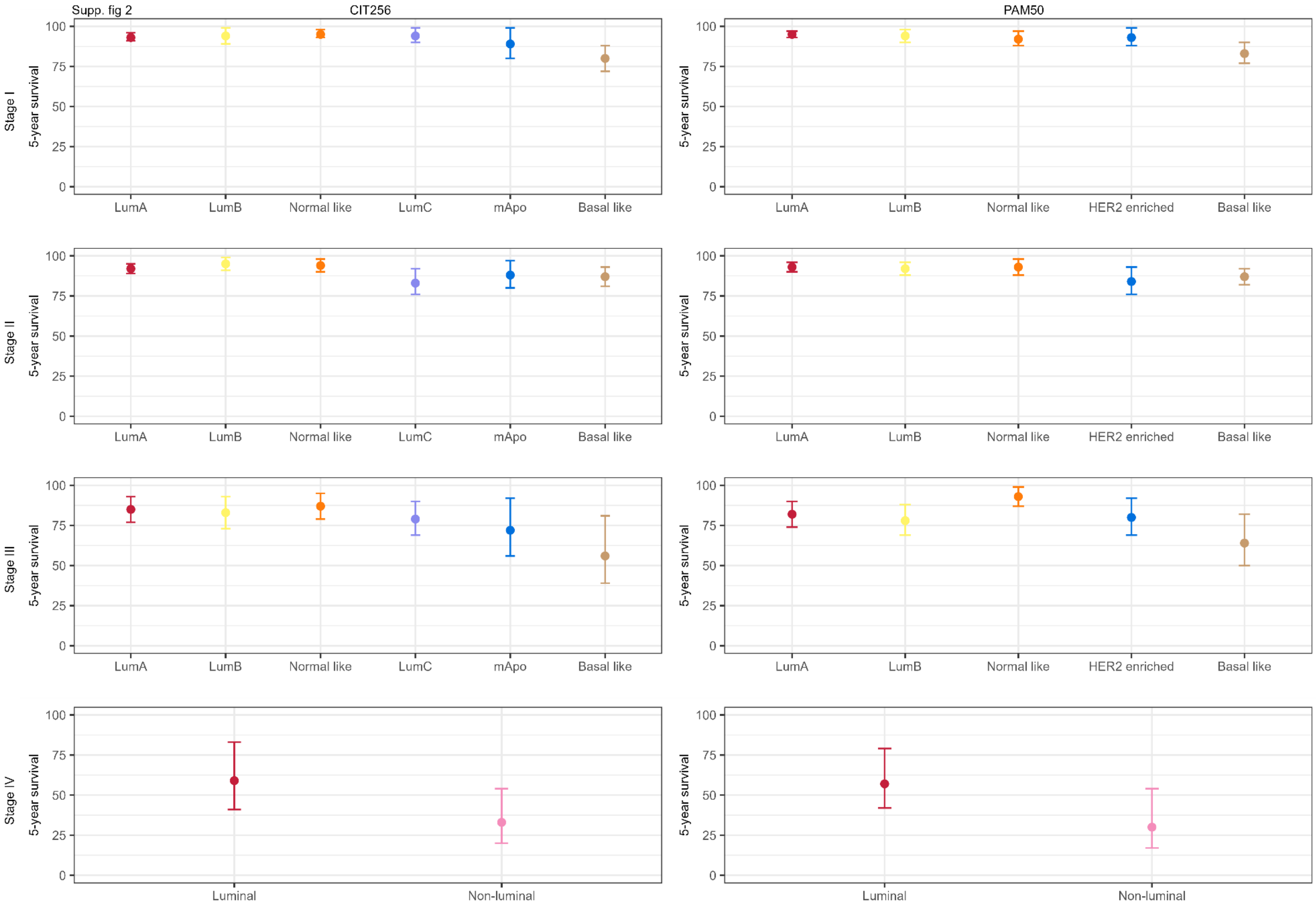

Supplement: Supplemental data [file jciinsight-9-178114-s072.pdf]
